# Germline *NF2* variant position constrains somatic second hits and determines clinical severity in Neurofibromatosis Type 2-related schwannomatosis

**DOI:** 10.64898/2026.07.27.26359036

**Published:** 2026-07-30

**Authors:** Niveditha Ravindra, David Asuzu, Emma Celano, Santosh Kumar, Arshi Chopra, Debjani Mandal, Dustin Mullaney, Dhruval Bhatt, Maxwell T Laws, Christina Hayes, Isac Kunnath, Bayu Sisay, Abdel Elkahloun, Ashok Ashthagiri, Tanya Lehky, Christopher Zalewski, John D Heiss, Hung J Kim, Prashant Chittiboina

## Abstract

**Purpose:**

Neurofibromatosis type 2-related schwannomatosis (NF2-SWN) is an autosomal dominant tumor syndrome with complete penetrance and variable expressivity. Underlying patterns of disease burden and severity are largely unexplained.

**Methods:**

We comprehensively phenotyped 168 NF2-SWN patients over a mean duration of 4.5 years. We used a custom sequencing panel of *NF2* and schwannomatosis genes to identify germline (n=166) and somatic variants in tumors (n=37). An optimized composite severity (CSS) score based on clinical and radiological data was created to analyze the effect of genetic variants on phenotype.

**Results:**

We found significant variable expressivity not explainable by demographic variables. Germline variants included premature termination (42%), splice-site (18%), and large deletions (16%). The CSS successfully predicted worsening clinical function in patients. Unsupervised clustering of clinical data revealed distinct phenotypic clusters that corresponded to CSS. Mosaicism, however, was not associated with CSS or any other disease severity marker. CSS was significantly associated with germline variant location along the *NF2* locus. Specifically, FERM-F1 and the α-helical variants were associated with increased disease severity. Within tumors, germline variants with severe effects on merlin acquired milder somatic second-hits at the *NF2* locus.

**Conclusion:**

We identified a second-hit modifier to the Mendelian first-hit: severe germline variants were associated with milder somatic variants, and vice versa. This phenomenon partly explains the variable expressivity in NF2-SWN.

**Context Summary:**

Neurofibromatosis type 2-related schwannomatosis (NF2-SWN) displays striking variability in tumor burden and clinical severity, but the biological basis for this heterogeneity has remained unclear. Using deep longitudinal phenotyping, whole-neuroaxis imaging, and integrated germline-somatic genomic analysis, we show that germline *NF2* variant position determines the allowable somatic ‘second hits’ that can give rise to tumors. These data support a merlin-dosage model in which tumorigenesis likely occurs only within an intermediate range of merlin function, bounded by insufficiently tumorigenic and lethal levels on each side. These findings shed light on long-standing inconsistencies in genotype-phenotype relationships in NF2-SWN and help provide a mechanistic basis for predicting disease severity.

## Full Text Availability

The license terms selected by the author(s) for this preprint version do not permit archiving in PMC. The full text is available from the preprint server.

